# Dried Ginger (*Zingiber officinalis*) Inhibits Inflammation in a Lipopolysaccharide-Induced Mouse Model

**DOI:** 10.1155/2013/914563

**Published:** 2013-06-27

**Authors:** You Yeon Choi, Mi Hye Kim, Jongki Hong, Sung-Hoon Kim, Woong Mo Yang

**Affiliations:** ^1^Department of Prescriptionology, College of Oriental Medicine, Institute of Oriental Medicine, Kyung Hee University, Seoul 130-701, Republic of Korea; ^2^College of Pharmacy, Kyung Hee University, Seoul 130-701, Republic of Korea; ^3^Cancer Preventive Material Development Research Center, College of Oriental Medicine, Kyung Hee University, Seoul 130-701, Republic of Korea

## Abstract

*Objectives*. Ginger rhizomes have a long history of human use, especially with regards to their anti-inflammatory properties. However, the mechanisms by which ginger acts on lipopolysaccharide-(LPS-)induced inflammation have not yet been identified. We investigated the anti-inflammatory effects of dried *Zingiber officinalis* (DZO) on LPS-induced hepatic injury. *Methods*. ICR mice were given a DZO water extract (100, 1000 mg/kg) orally for three consecutive days. On the third day, they were administered by LPS intraperitoneally. To investigate the anti-inflammatory effects of DZO, histological, cytokine expression, and protein factor analyses were performed. *Results*. Oral administration of DZO significantly reduced pathological changes in the liver and proinflammatory cytokines including interferon-(IFN-)**γ** and interleukin-(IL-)6 in the serum. In addition, DZO inhibited LPS-induced NF-**κ**B activation by preventing degradation of the I**κ**B-**α**, as well as the phosphorylation of ERK1/2, SAPK/JNK, and p38 MAPKs. These were associated with a decrease in the expression of inducible nitric oxide synthase (iNOS) and cyclooxyenase-2 (COX-2). *Conclusions*. Our data provide evidence for the hepatoprotective mechanisms of DZO as an anti-inflammatory effect. Furthermore, use of DZO to treat could provide therapeutic benefits in clinical settings.

## 1. Introduction 

The liver is well known to play an important role in the initiation of multiorgan failure, the most lethal complication in the inflammatory response [1]. In particular, severe liver injury results from the massive death of liver cells leading to severe impairment of liver function [2]. Microbes and their virulence factors enter the hepatic circulation where they first activate Kupffer cells to produce proinflammatory mediators, including necrosis factor-(TNF-)*α*, interleukin, (IL-)1, IL-6 and eicosanoids [3]. These mediators cause not only microbial killing but also structural and functional liver damage concerning mainly the parenchymal cells [4].

Lipopolysaccharide (LPS), the major constituent of the outer cell wall of Gram-negative bacteria, has been widely used to examine the mechanisms of inflammation that produce typical hepatic necrosis followed by fulminant hepatic failure [5]. It has been reported that under LPS stimulation, Kupffer cells release pro-inflammatory cytokines [6]. In addition, activation of LPS-induced nuclear factor-kappa B (NF-*κ*B) mediates the mitogen-activated protein kinases (MAPKs) and subsequently regulates cyclooxygenase-(COX-)2 expressions, as well as inducible nitric oxide synthases (iNOS) expressions [7]. Furthermore, COX-2 and iNOS expression have been proven to contribute to inflammatory disease [8]. Therefore, these cytokines represent an ideal target for neutralization of LPS [9]. Currently, long-term use of anti-inflammatory drugs is associated with side effects such as fever, facial flushing, and aching muscles. Therefore, using a natural product to treat inflammatory diseases may be more effective and have fewer side effects [10]. 

Ginger rhizome (*Zingiber officinale *Roscoe, *Zingiberaceae*) has long been used in the world as a popular spice food as well as a medicinal herb because of its high content of antioxidants and anti-inflammatory properties [11, 12]. Ginger rhizome comprises various kinds of chemicals including 6- and 8-series of gingerols and shogaols, among which gingerol is the major ingredient representing a variety of bioactivities including antitumor promotional and antiproliferative [13]. Studies by Nonn et al. have shown that 6-gingerol inhibited the TNF-*α*, and IL-1*β*-induced increase in the p38-dependent NF-*κ*B activation and expression of pro-inflammatory genes of IL-6 and IL-8 in normal prostatic epithelial cells [14]. Extensive studies in recent years have displayed that 6-gingerol of ginger metabolites inhibits COX-2 expression by blocking the activation of p38 MAP kinase and NF-*κ*B in phorbol ester-stimulated mouse skin [15]. 6-Shogaol suppressed LPS-induced up-expression of iNOS and COX-2 in murine macrophages [16]. These effects may be due to the pharmacological activity of biologically active compounds involved in reducing inflammation [17]; however, the anti-inflammatory mechanisms of DZO on LPS-induced liver damage are not yet fully understood.

 This study addressed the question of whether DZO has a hepatoprotective effect in LPS-induced inflammations. We also attempted to understand the mechanism by which DZO can reduce LPS-induced inflammation as well as hepatic failure.

## 2. Materials and Methods

### 2.1. Chemicals and Regents

LPS (*Escherichia coli* 0111:B4) was purchased from Sigma-Aldrich (BD Bioscience, USA). Mouse IFN-*γ*, IL-6, and the TMB substrate reagent ELISA kit were purchased from BD Bioscience (San Jose, CA, USA). RIPA buffer and protease inhibitor cocktails were obtained from Roche (Indianapolis, IN, USA). Dual-color protein standards, protein assays, Tween-20, acrylamide, ammonium persulfate, skim milk, enhanced chemiluminescence (ECL) detection reagent, and PVDF membranes were purchase from Bio-Rad Laboratories (Hercules, USA). Rabbit antibeta-actin, iNOS, COX-2, NF-*κ*B, I*κ*B-*α*, phosphor-I*κ*B-*α*, and anti-rabbit alkaline phosphatase-conjugated secondary antibody were purchased from Santa Cruz Biotechnology, Inc. (Santa Cruz, CA, USA). Anti-ERK1/2, phospho-ERK1/2, anti-SAPK/JNK, phospho-SAPK/JNK, anti-p38, and phospho-p38 MAPK were purchased from Cell Signaling Technology Inc. (Beverly, MA, USA).

### 2.2. Preparation of DZO

Dried *Zingiber officinalis* (DZO) was purchased from Omni Herb Inc. (Andong-si, Gyeongbuk, Republic of Korea). DZO extract was prepared by decocting 250 g dried herb with 5 L boiling distilled water for 1 h 30 min. The filtrate was concentrated under reduced pressure and lyophilized. After filtration, an aqueous solution of the extract was concentrated in a rotary evaporator, freeze dried for 3 days, and stored at 4°C until used. The yield of DZO was approximately 10.45% w/w (dried weight 26.12 g). A voucher specimen (DZO001) was deposited at our laboratory. Before each experiment, DZO extract was dissolved in distilled water and vortexed for 2 min at room temperature.

### 2.3. HPLC Analysis of Standards to DZO

HPLC analyses were carried out with an Agilent Series 1100 HPLC system (Palo Alto, CA, USA) consisting of a quaternary delivery system, an autosampler, and a diode array detector (DAD). The chromatographic separation analysis was carried out on a Shiseido UG 120 C18 (250 × 4.6 mm, i.d., 5 *μ*m) column. The mobile phases consisted of solvent A (acetonitrile, ACN) and solvent B (water). The standard materials used for the quantitative analysis of DZO were gingerol and shogaol. A gradient program was performed: 0–12 min (40–50% A); 12–24 min (50–70% A); 24–30 min (70–100% A); 30–40 min (100% A), back to the initial conditions for equilibration. UV detection wavelength was set at 220 nm. The flow rate and injection volume were set at 1 mL/min and 10 *μ*L, respectively, at room temperature (25°C).

### 2.4. Animal Treatment and Induction of Inflammation

Female ICR mice (7 weeks old, weighing 28–30 g) were obtained from Japan SLC Inc. (Hamamatsu, Japan). The mice were kept in sterilized cages (*n* = 5) under standard conditions (12 h light and 12 h dark), at 24 ± 2°C with a relative humidity of 40–80%. They were fed a laboratory diet, water was provided *ad libitum*, and they were housed for 7 days before experimentation. They were arbitrarily divided into four groups: normal (control group; no treatment), LPS (a negative control group; treated with LPS 35 mg/kg), LPS + DZO100 (treated with LPS and DZO100 mg/kg), and LPS + DZO1000 mg/kg (treated with LPS and DZO1000). After the 7-day adaptation period, mice in groups 3 and 4 were orally administered by DZO in distilled water for three consecutive days, while groups 1 and 2 received an equivalent volume of water as the control. On day 3, 1 h after DZO administration, all animals in groups 2, 3, and 4 were intraperitoneally injected with LPS dissolved in distilled water. Blood from suborbital and liver samples was collected 6 h after the LPS challenge. All experiments were conducted according to the guidelines of the Committee on Care and Use of Laboratory Animals of the Kyung Hee University. (KHUASP(SE)-12-020).

### 2.5. Histological Assays

Liver tissue was fixed in 10% buffered formaldehyde, embedded in paraffin for 24 h, and serially sectioned to a thickness of 5 *μ*m. Sections were stained with hematoxylin and eosin (H&E) and examined for general morphology. Pathological changes were evaluated under a light microscope using the Leica Application Suite (LAS; Leica Microsystems, Buffalo Grove, IL, USA). Digital images were taken at a magnification of ×100 and ×400.

### 2.6. Determination of Cytokine Levels (IFN-*γ* and IL-6)

Serum samples were obtained from centrifuged blood (14000 ×g, 30 min) and stored at −80°C until needed. IL-6 and IFN-*γ* concentrations were measured using a mouse TNF-*α*, IL-4, IL-6, and IFN-*γ* ELISA kit (BD Bioscience, San Jose, CA, USA), according to the manufacturer's instructions. The optical density of each well was read on an ELISA Reader (Molecular Devices, Downingtown, PA, USA) using 450 nm and 570 nm filters.

### 2.7. Preparations of Protein Extracts

Liver tissue was frozen in liquid nitrogen and then homogenized. For the cytoplasmic extracts, 100 mg frozen liver tissue homogenate (*n* = 5 in each group) was incubated on ice for 15 min in cytoplasmic buffer (10 mM HEPES, pH 7.9, 10 mM KCl, 0.1 mM EDTA, 0.1 mM EGTA, 1 mM DTT, 0.15% Nonidet P-40, 50 mM *β*-glycerophosphate, 10 mM NaF, and 5 mM Na_3_VO_4_) and the protease inhibitor cocktail. The resulting homogenate was centrifuged at 12000 ×g for 30 min at 4°C and the supernatant was carefully removed without disturbing the pellet. The supernatant was used to detect activated I*κ*B-*α* and phospo-I*κ*B-*α*. To extract the nuclear proteins, nuclear buffer (20 mM HEPES, pH 7.9, 400 mM NaCl, 1 mM EDTA, 1 mM EGTA, 1 mM DTT, 0.50% Nonidet P-40, 50 mM *β*-glycerophosphate, 10 mM NaF, and 5 mM Na_3_VO_4_, containing the protease inhibitor cocktail) was added to the pellet and then incubated on ice for 15 min. This was centrifuged at 12000 ×g for 15 min at 4°C to determine the NF-*κ*B. Levels of iNOS, COX-2, and MAPKs (ERK1/2, SAPK/JNK, p38) were confirmed using whole protein extracts. Liver tissue (*n* = 5 in each group) was homogenized on ice for 15 min in RIPA buffer (50 mM Tris-HCl, pH 7.4, 1% Nonidet P-40, 0.5% sodium deoxycholate, 150 mM NaCl) and the protease inhibitor cocktail. The homogenate was incubated on ice for 15 min after vortexing, and this process was repeated four times. Finally, the homogenate was centrifuged at 10,000 ×g for 30 min at 4°C, and the supernatant was analyzed for the whole protein extracts.

### 2.8. Determination of NF-*κ*B, I-*κ*B, MAP Kinase (ERK1/2, SAPK/JNK, p38), COX-2, and iNOS

Samples (40 *μ*g) of protein from each liver homogenate (nuclear, cytoplasmic, and whole fraction) were loaded onto 15% polyacrylamide gels for electrophoresis. Then, the proteins were transferred to polyvinylidene fluoride (PVDF) membranes and incubated at room temperature for 60 min in TBS buffer containing 0.1% Tween (TBS-T) and 5% dried skim milk to block nonspecific binding. The membrane was incubated overnight with one of the primary antibodies (antibodies; *β*-actin, NF-*κ*B, phospho-I*κ*B-*α*, ERK1/2, phospho-ERK, SAPK/JNK, phospho-SAPK/JNK, p38, phospho-p38, iNOS, and COX-2; dilution 1 : 1000 in TBS-T). Anti-rabbit alkaline phosphatase-conjugated secondary antibody (dilution 1 : 2000 in TBS-T) was used to detect the target proteins. After incubation for 2 h at room temperature, blots were detected using an enhanced chemiluminescence (ECL) detection reagent. *β*-Actin was used as an internal loading control. The relative band density was calculated with respect to the *β*-actin blot developed under similar conditions using a computerized densitometry system.

### 2.9. Statistical Analysis

All values are expressed as means ± SD. Differences between the mean values of normally distributed data were assessed by one-way ANOVA (Dunnett's *t* test) and Student's *t* test. Statistical significance was accepted at *P* < 0.05.

## 3. Results

### 3.1. Phytochemical Analyses of Standard Materials to DZO Extract

**Figure 1:**
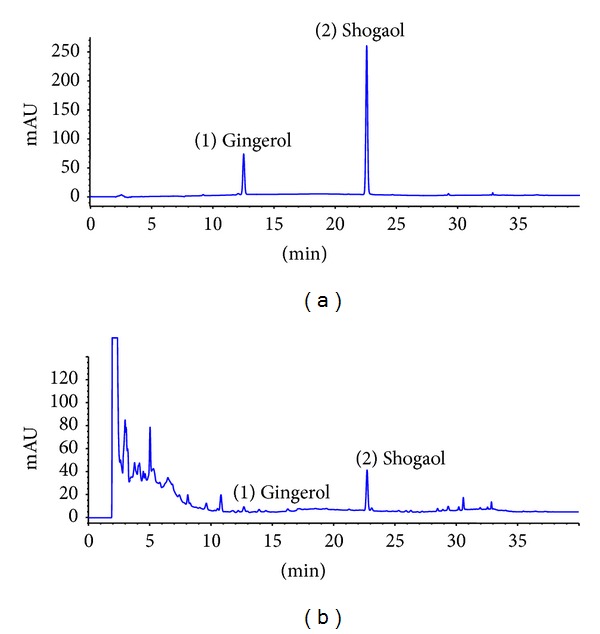
HPLC chromatogram of standard materials (a) and DZO (b). Two external standards were used for HPLC analysis.

HPLC was used for detection of DZO constituent research with gingerol and shogaol as standard materials. Two peaks on DZO were synchronized with gingerol and shogaol, which are components of *Zingiber officinalis *(Figure 1).

### 3.2. DZO Attenuates Inflammatory Responses in LPS-Induced Hepatic Failure

**Figure 2:**
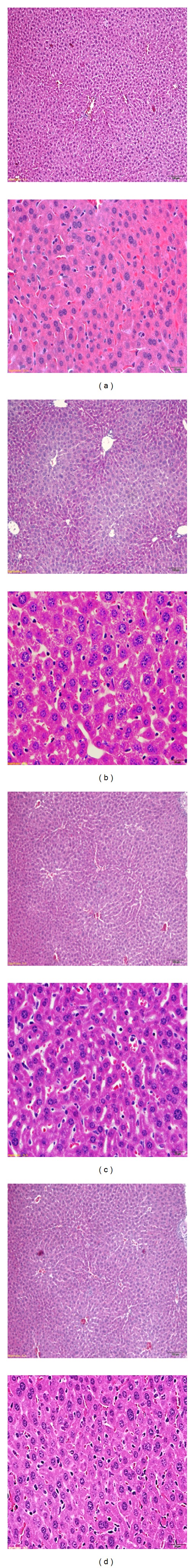
Effects of two doses of DZO on histopathological changes in liver tissues of mice treated with LPS. (a) Normal group: liver tissue structure showed no pathological abnormalities. (b) LPS group: liver tissue structure showed apparent broad hemorrhagic necrosis. (c) LPS + DZO group (100 mg/kg): liver tissue structure showed minimal hepatocellular necrosis. (d) LPS + DZO group (1000 mg/kg): liver tissue structure was similar to normal group. Typical images were chosen from each experimental group (×100 in the upper panel and ×400 in the lower panel).

Liver architecture and structure were examined for five specimens taken from each of the four treatment groups (normal, LPS, DZO100, and DZO1000). Histological analysis indicated normal liver architecture and structure in the normal control group (Figure 2(a)). However, LPS-induced livers showed broad hemorrhagic necrosis and extensive areas of portal inflammation (Figure 2(b)). These pathological changes were less severe in DZO treatment groups (Figures 2(c) and 2(d)).

### 3.3. Effects of DZO on Serum Levels of IFN-*γ* and IL-6 in LPS-Induced Inflammation

**Figure 3:**
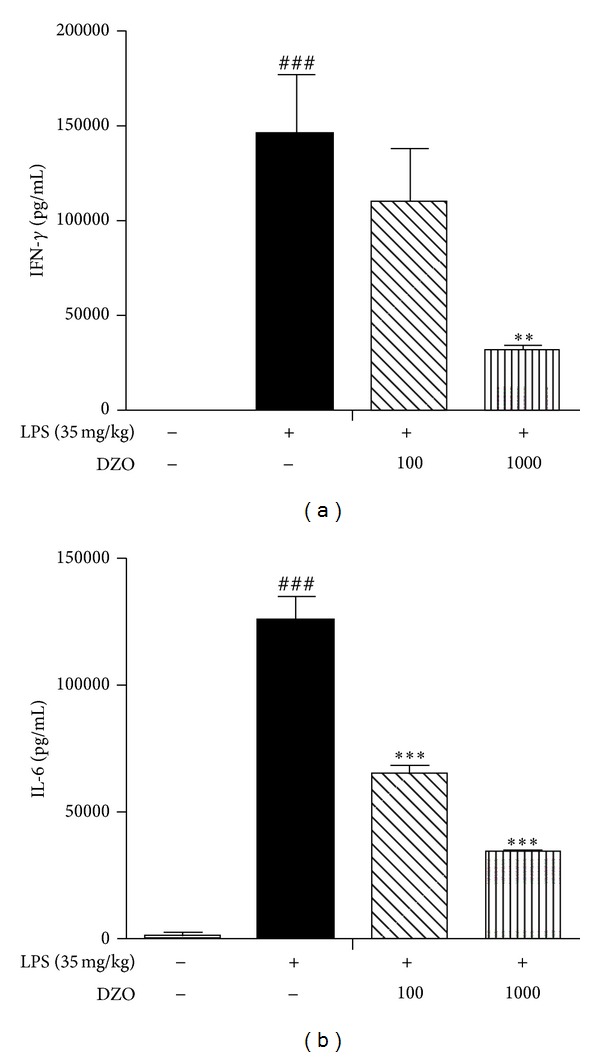
Effect of two doses of DZO on IFN-*γ* (a) and IL-6 (b) levels in the serum of mice treated with LPS. The results are presented as the mean ± SEM (*n* = 5). The serum levels of IFN-*γ* and IL-6 in DZO-treated group (100 or 1000 mg/kg) were significantly attenuated. ^#^Indicates significance for the difference between normal control group and LPS group (***P < 0.001). *Indicates significant difference from LPS group (***P < 0.001, **P < 0.01).

The regulatory effects of DZO (100, 1000 mg/kg) on the systemic secretion of circulating cytokines, such as IFN-*γ* and IL-6, were examined by ELISA. Serum levels of IFN-*γ* and IL-6 significantly increased 6 h after LPS challenge compared to the normal group. In contrast, serum levels of IFN-*γ* and IL-6 in DZO (100, 1000 mg/kg) treatment groups were significantly lower (Figure 3).

### 3.4. DZO Reduces the Expression of NF-*κ*B and I*κ*B-*α* in LPS-Induced Inflammation

**Figure 4:**
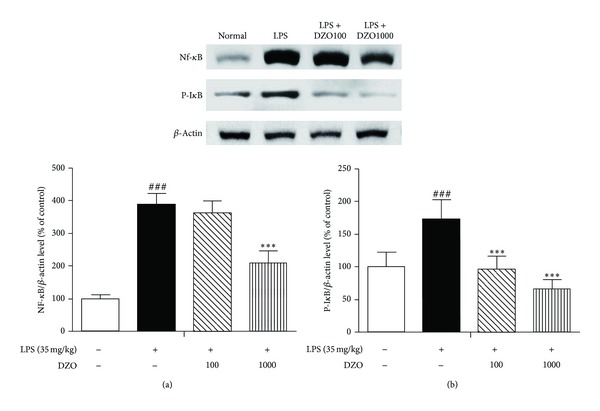
Effects of two doses of DZO on the activation of NF-*κ*B (a) and I*κ*B-*α* (b) in the liver of mice treated with LPS. DZO inhibited the degradation of I*κ*B-*α* and NF-*κ*B nuclear translocation. Similar results were obtained in three independent experiments, and the results obtained from one of three representative experiments are shown. ^#^Indicates significance for the difference between normal control group and LPS group (***P < 0.001). *Indicates significant difference from LPS group (***P < 0.001).

To understand the mechanisms of DZO inhibition of the pro-inflammatory cytokines, we investigated whether DZO inhibits both the degradation of I*κ*B-*α* and the nuclear translocation of NF-*κ*B. DZO (100, 1000 mg/kg) significantly suppressed the LPS-induced degradation of I*κ*B-*α* (Figure 4(a)) as well as the translocation of NF-*κ*B (Figure 4). It also inhibited LPS-induced phosphorylation of I*κ*B-*α* in the cytoplasm.

### 3.5. DZO Reduces the Expression of MAP Kinase (ERK1/2, SAPK/JNK, and p38) in LPS-Induced Inflammation

**Figure 5:**
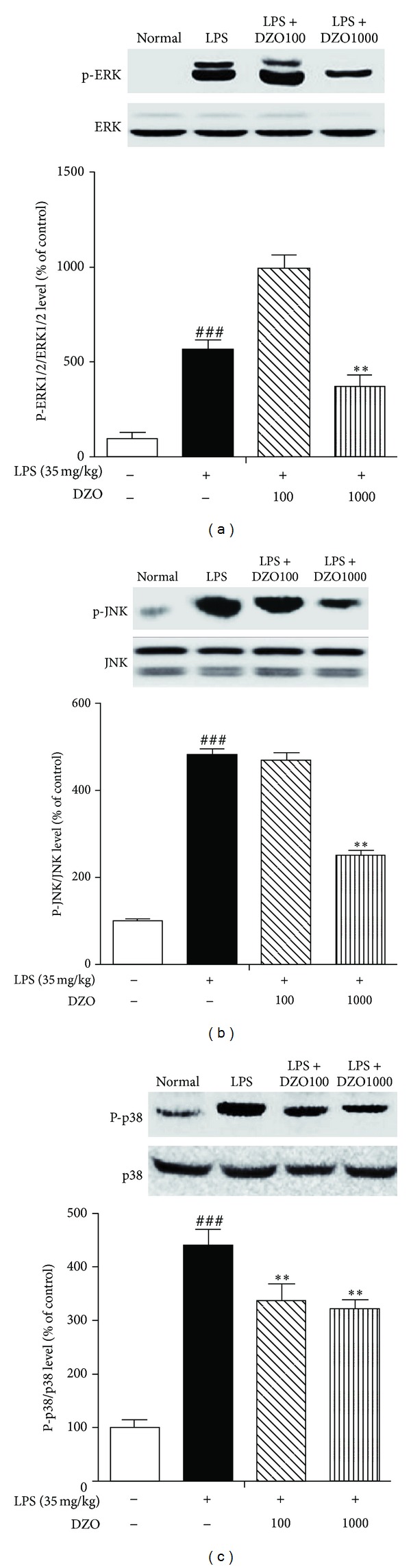
Effects of two doses of DZO on the phosphorylation of MAPKs (ERK1/2 (a), SAPK/JNK (b), and p38 MAPKs (c)) in the liver of mice treated with LPS. DZO remarkably attenuated LPS-induced phosphorylation of ERK1/2, SAPK/JNK, and p38 MAPKs. Similar results were obtained in three independent experiments, and the results obtained from one of three representative experiments are shown. ^#^Indicates significance for the difference between normal control group and LPS group (***P < 0.001). *Indicates significant difference from LPS group (**P < 0.01).

The effects of DZO on LPS-induced phosphorylation of ERK1/2, SAPK/JNK, and p38 MAPKs were examined by western blot. DZO markedly reduced LPS-induced phosphorylation of all compounds, whereas the nonphosphorylated forms used as a control remained the same (Figure 5). Thus, DZO might suppress the production of LPS-induced pro-inflammatory mediators by inhibiting phosphorylation of the signaling proteins.

### 3.6. DZO Inhibits the Expression of iNOS and COX-2 in LPS-Induced Inflammation

**Figure 6:**
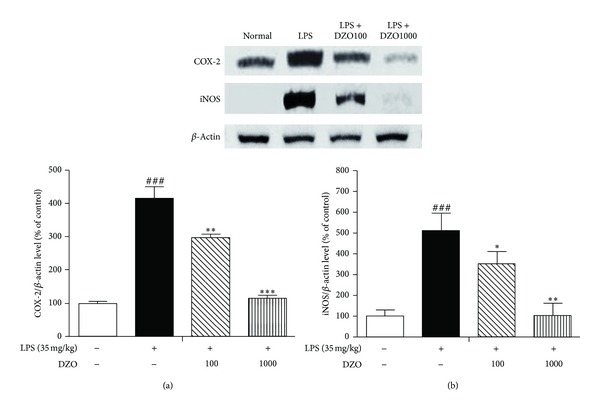
Effects of two doses of DZO on the expression of COX-2 (a) and iNOS (b) in the liver of mice treated with LPS. The expression of COX-2 and iNOS was increased significantly after exposure to LPS for 6 h, and this effect was blocked by pretreatment with DZO at a dose of 100 or 1000 mg/kg. ^#^Indicates significance for the difference between normal control group and LPS group (***P < 0.001). *Indicates significant difference from LPS group (***P < 0.001, **P < 0.01, *P < 0.05).

The inflammatory factors iNOS and COX-2 are correlated with LPS stimulation. We tested the anti-inflammatory effects of DZO on LPS-induced iNOS and COX-2 expression, by western blot. Both factors increased significantly in the LPS-induced groups, but DZO inhibited their expression in the treatment groups (100, 1000 mg/kg) (Figure 6).

## 4. Discussion

We demonstrated that pretreatment with DZO significantly reduced the inflammatory response of LPS-induced inflammation. LPS induced liver-related inflammation in a mouse model of viral hepatitis that closely resembles human viral hepatitis [18]. A previous study reported that LPS-induced histological changes showed lymphocyte and neutrophil infiltration increasing in the central and portal areas [19]. Our histological analysis showed the inhibitory effects of DZO on necrotic hepatocytes and tissue damage with excessive production of inflammatory cytokines in the LPS-induced liver and serum. In particular, DZO1000 group largely attenuated LPS-induced broad hemorrhagic necrosis of liver structure similar to normal group. These results indicate that DZO is effective for controlling the response of LPS-induced liver damage.

Several studies have indicated that the inflammatory response to LPS challenge is associated with the release of pro-inflammatory cytokines such as IFN-*γ* and IL-6 [20]. In addition, mediators generated by LPS stimulation assist the innate immune response, but their overproduction results in acute inflammation that can cause tissue injury and organ failure [21]. Here, we confirmed that DZO inhibits the expression of LPS-induced IFN-*γ* and IL-6, which are significantly elevated in LPS-induced inflammation. These results indicate that DZO might suppress the inflammatory response via the inhibition of inflammatory cytokines supporting our histological analysis. 

The transcription regulator NF-*κ*B plays a pivotal role in activating subsequent signaling pathways, especially the regulation of pro-inflammatory molecules [22]. Also, activation of LPS-induced NF-*κ*B causes phosphorylation of I*κ*B-*α* kinase (IKK), leading to degradation of I*κ*B-*α* and translocation of NF-*κ*B into the nucleus [23]. These studies may provide a target to specifically downregulate the expression of NF-*κ*B with inhibition of I*κ*B-*α* degradation. In this study, DZO inhibited LPS-induced NF-*κ*B transcription activity as well as I*κ*B-*α* protein expression in the liver compared to negative control group.

Many studies have reported that MAPKs mediate the activation of the transcription factor NF-*κ*B [24]. To explore the mechanisms of NF-*κ*B inactivation by DZO, the effects of DZO on LPS-induced phosphorylation of the Erk1/2, SAPK/JNK, and p38 MAP kinases were examined. All of the kinases were overexpressed after exposure to LPS, but that expression was reduced after DZO exposure. This suggests that the hepatoprotective activity of DZO is due to NF-*κ*B inhibition via the inhibition of LPS-induced phosphorylation of MAPKs. 

Furthermore, NF-*κ*B activation mediates the expression of rapid-response genes, including pro-inflammatory mediators such as iNOS and COX-2 [25]. The present study confirmed that LPS stimulates iNOS and COX-2, which was associated with overexpression of NF-*κ*B, whereas orally administrated DZO greatly reduces the expression of iNOS and COX-2 and hence the expression of NF-*κ*B. It is likely that the anti-inflammatory activity of DZO contributes to the reduced expression of iNOS and COX-2 in LPS-induced liver injury. Also, proinflammation cytokines, IL-6 and TNF-*α* are upregulated by the expression of COX-2 and iNOS [26]. Taken together, these data suggest that DZO inhibits the expression of iNOS and COX-2 through inactivation of NF-*κ*B by reducing I*κ*B-*α* phosphorylation. We assume that the hepatoprotective effects of DZO may be due to the anti-inflammatory compounds such as gingerols and shogaols. These results are helpful in understanding the anti-inflammations properties of DZO. 

## 5. Conclusion

We found that DZO inhibits LPS-induced inflammation via regulation of NF-*κ*B and MAP kinases. DZO significantly inhibits the production of IFN-*γ* and IL-6 and suppresses NF-*κ*B by degradation of I*κ*B-*α*. These activities appear to be mediated via downregulation of the ERK1/2, SAPK/JNK, and p38 MAP kinases signaling pathways and suppression of iNOS and COX-2. Our data provide evidence for a mechanism by which DZO acts as an anti-inflammatory agent. Strategic use of DZO in treating inflammatory diseases could provide therapeutic benefits for future clinical use.
